# Western lifestyle as a driver of dysbiosis in colorectal cancer

**DOI:** 10.1242/dmm.049051

**Published:** 2021-06-01

**Authors:** Nora J. Foegeding, Zachary S. Jones, Mariana X. Byndloss

**Affiliations:** 1 Department of Pathology, Microbiology and Immunology, Vanderbilt University Medical Center, Nashville, Tennessee 37232, USA; 2 Vanderbilt Institute for Infection, Immunology and Inflammation, Vanderbilt University Medical Center, Nashville, Tennessee 37232, USA

## Abstract

Landmark discoveries in the gut microbiome field have paved the way for new research aimed at illuminating the influence of microbiota in colorectal cancer. A major challenge is to account for the effect of inherently variable environmental factors on the host and the gut microbiome, while concurrently determining their contribution to carcinogenesis. Here, we briefly discuss the role of the gut microbial community in colorectal cancer and elaborate on the recent insight that environmental factors related to a Western diet and lifestyle may drive the bloom of tumorigenic members of the gut microbiota. We also discuss how future research focused on untangling host-microbe interactions in the colon may influence medical insights that relate to the prevention and treatment of colorectal cancer.

## Abstract (precis)

**Summary:** This Perspective explores the mechanistic insights and future research directions connecting Western lifestyle-induced changes in colonic epithelial physiology with the outgrowth of tumorigenic bacteria that contribute to colorectal cancer pathogenesis.

## Introduction

Residing within the gastrointestinal tract is a dynamic microbial ecosystem – an assembly of bacteria, archaea, fungi, protists and viruses – that collectively functions as an integral part of human biology. Numerous studies have linked alterations in gut microbiota composition, termed dysbiosis, to an assortment of health conditions and diseases. Although much effort has gone into cataloging the composition of gut microbiota and their genes (the gut microbiome) during states of health and disease, a major focus of future research should be directed towards a mechanistic understanding of how host-associated microbial communities contribute to disease. Deeper insight into these relationships will reveal strategies by which host-microbe interactions may be modulated to prevent disease onset and progression, and to influence response to therapy.

Here, we consider the role of the gut microbiome in colorectal carcinogenesis, with a focus on the recent insight that a Western lifestyle – particularly the consumption of a high-fat diet and regular exposure to antibiotics – can drive the expansion of tumorigenic members of the gut microbiota. We believe that factoring host-microbe interactions into colorectal cancer (CRC) research will influence medical insights relating to CRC prevention and treatment.

## Gut microbiota and gastrointestinal health

Pioneering discoveries over the last decade have started to uncover mechanisms by which gut microbiota benefit host health. One prominent example is the production of short-chain fatty acids (SCFAs) during bacterial fermentation of indigestible oligosaccharides. Supplied to humans only by bacteria, SCFAs are an essential energy source for colonocytes (Roediger, 1980, 1982) and influence regulatory T cell homeostasis (Furusawa et al., 2013; Smith et al., 2013), crucial for limiting intestinal inflammation.

However, defining the impact of the gut microbiome on human health is an inherently complex task, as the composition of gut microbiota is not static but always in flux. Some of the most drastic changes in the structure of gut microbiota occur as humans develop from infants to adults (Yatsunenko et al., 2012). Throughout the first year of life, milk plays a central role in shaping the gut microbial community of breast-fed infants (Kirmiz et al., 2018). This is evidenced by the early-life gut microbiota being dominated by *Bifidobacterium*, which metabolize the indigestible human milk oligosaccharides (HMOs) present in breast milk, as well as by lactic acid bacteria, i.e. *Streptococcus*, *Lactococcu*s and *Lactobacillus,* which digest another milk component – lactose (Yatsunenko et al., 2012; Dominguez-Bello et al., 2019). As infants begin consuming solid food, bacterial diversity steadily increases and, by about three years of age, the composition of the gut microbiome begins to reflect that of the adult (Dominguez-Bello et al., 2019; Yatsunenko et al., 2012).

Once adulthood has been reached, an individual's gut microbiome is relatively stable, yet still at the mercy of rapidly responding to cues from other microbes, host cells and the unceasing supply of food and xenobiotics passing through the gastrointestinal tract. Indeed, dietary factors (David et al., 2014), food additives (Chassaing et al., 2015), antibiotics (Byndloss et al., 2017), medications (Maier et al., 2018) and other environmental factors have been shown to profoundly shape gut microbial communities. There are numerous mechanisms by which environmental factors spur fluctuations in the composition of gut microbiota, including by serving as nutrient sources (Sonnenburg et al., 2016), sequestering normally abundant elements (Stacy et al., 2021) and depleting specific microbes (Byndloss et al., 2017). In addition, environmental factors can indirectly affect the gut microbial composition. For example, the ability of antibiotics to increase the inflammatory ‘tone’ in the colon creates a favorable environment for the outgrowth of pathogens (Byndloss et al., 2017). Although the blueprints for a healthy gut microbiome are not-yet-known, it is clear that many pathologies are linked to changes in host-associated microbial communities.

Considering the close proximity and constant crosstalk between the colorectal epithelium and gut microbiota, it is perhaps expected that gut microorganisms affect colorectal carcinogenesis.

## Gut microbiota and CRC

Worldwide, CRC is the second leading cause of cancer-related death (Sung et al., 2021), and clinicians are reporting a startling increase in the incidence of early-onset disease, generally defined as CRC diagnosed in individuals less than 50 years of age (Sung et al., 2019). Although genetic predisposition is a leading risk factor for CRC, inherited mutations are estimated to account for ∼35% of all cases (Lichtenstein et al., 2000). The remaining 65% of CRCs are sporadic, indicating that environmental factors have a principal role in triggering colorectal carcinogenesis (Lichtenstein et al., 2000).

Considering the close proximity and constant crosstalk between the colorectal epithelium and gut microbiota, it is perhaps expected that gut microorganisms affect colorectal carcinogenesis. Sequencing studies have not only mapped consistent changes of the gut microbiota composition in CRC patients compared with healthy individuals (Wirbel et al., 2019) but have also begun to catalog microbial signatures associated with stages of CRC development (Nakatsu et al., 2015; Yachida et al., 2019). The gut microbiota can enhance CRC development through its ability to foster a pro-inflammatory environment, with recognition of bacterial components by the innate and adaptive immune system leading to the production of cytokines and other inflammatory products (Carvalho et al., 2012; Gronbach et al., 2014). The impact of commensal gut bacteria on the initiation and development of CRC also occurs through direct effects of bacterially derived metabolites and virulence factors. Three prominent examples of gut microbial toxins thought to potentiate colorectal carcinogenesis include the *Bacteroides fragilis* toxin (Wu et al., 2009)*,* the *Fusobacterium nucleatum* adhesion protein A (Rubinstein et al., 2013) and colibactin produced by some *Escherichia coli* strains (Arthur et al., 2012)*.* These bacterial toxins have gained attention for their ability to promote colorectal carcinogenesis in animal models (Wu et al., 2009; Rubinstein et al., 2013; Arthur et al., 2012). Colibactin is a unique example within this group, as it is a genotoxin that can bind to DNA (Wilson et al., 2019) – thereby yielding a DNA adduct and inducing double-strand DNA breaks (Nougayrede et al., 2006) – indicating that it plays a role in the initiation of CRC. Colibactin is encoded by a gene cluster that is referred to as the polyketide synthase (*pks*) island (Nougayrede et al., 2006), and *pks*-positive (*pks*^+^) *E. coli* are detected more frequently in patients with CRC compared to healthy individuals (Dejea et al., 2018; Buc et al., 2013; Wirbel et al., 2019; Arthur et al., 2012).

With the increasing recognition of the gut microbiota contributions to systemic health, future CRC research will benefit from *in vivo* studies that delineate how environmental factors affect not only the host but also the gut microbiome, and how host-microbiome interactions contribute to cancer onset and progression in the colon.

With the increasing recognition of the gut microbiota contributions to systemic health, future CRC research will benefit from *in vivo* studies that delineate how environmental factors affect not only the host but also the gut microbiome, and how host-microbiome interactions contribute to cancer onset and progression in the colon. Such insight will reveal how modifiable risk factors, such as diet and lifestyle habits, exert synergistic detrimental effects on both the host and the microbiota, and inform on preventative interventions.

## Gut microbiota composition is regulated by the host

Whereas a growing list of sequencing and functional studies suggests a significant role for the microbiota in CRC, it remains unclear what initially drives the outgrowth of tumorigenic microbes in the gut. At homeostasis, obligate anaerobes make up the majority of gut microbiota, primarily *Firmicutes* and *Bacteroidetes* (Backhed et al., 2005). Being the main producers of SCFAs, obligate anaerobes are crucial to host nutrition (den Besten et al., 2013), immune development (Smith et al., 2013; Furusawa et al., 2013) and colonization resistance (Byndloss et al., 2017; Rivera-Chávez et al., 2016). By contrast, substantial shifts in gut microbiota composition associated with disease are often characterized by a sustained increase in the abundance of facultative anaerobes, including Enterobacteriaceae (Shin et al., 2015). Notably, *pks*^+^ *E. coli* are member species of the family Enterobacteriaceae and, as discussed above, are implicated in colorectal carcinogenesis (Arthur et al., 2012). Thus, perturbations in the host mechanisms that keep gut microbiota in check are likely to regulate the outgrowth of *pks^+^ E. coli* and, potentially, other microorganisms associated with CRC.

**Fig. 1.**
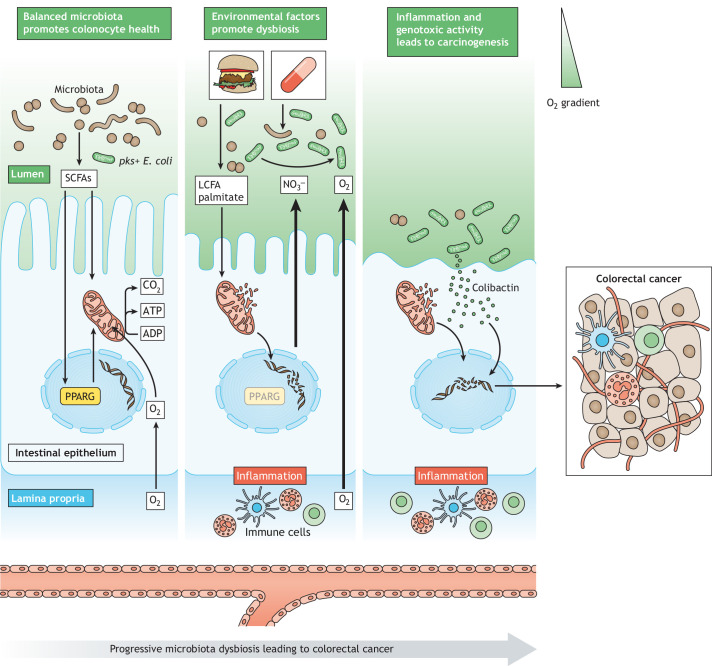
**The collective impact of a Western lifestyle and accompanying dysbiosis on the colorectal epithelium.** In a healthy colonic environment, the gut microbiota is dominated by obligate anaerobic bacteria (brown). Short-chain fatty acid (SCFA) production by obligate anaerobes activates PPARG and drives mitochondrial β-oxidation in colonocytes, supporting an anaerobic environment in the colonic lumen. By contrast, exposure to antibiotics and an obesogenic high-fat diet alters microbiota composition, impairs mitochondrial function in colonocytes and induces inflammation. Collectively, this permits an increase in the bioavailability of oxygen (O_2_) and nitrate (NO_3_^−^) in the colonic lumen. In turn, facultative anaerobic bacteria, such as *pks*^+^ *E. coli* (green), bloom due to their ability to utilize O_2_and NO_3_^−^ for respiration. Colorectal carcinogenesis may result from the combined impact of the increased inflammatory tone and DNA damage induced by mitochondrial dysfunction as well as the *pks*^+^ *E. coli* genotoxin colibactin.

The human immune system has a well-recognized role in maintaining a symbiotic relationship between the gut microbiota and the host (Zheng et al., 2020). In addition, recent insights suggest that colonic epithelial cells play a central role in shaping the structure of gut microbiota (Byndloss et al., 2017; Gillis et al., 2018). Colonocytes must maintain a highly oxidative metabolic state owing to the expression of elevated levels of peroxisome proliferator-activated receptor gamma (PPARG), a nuclear receptor that activates mitochondrial β-oxidation of fatty acids (Duszka et al., 2017). PPARG signaling in intestinal epithelial cells is activated by butyrate (Alex et al., 2013), a microbial-derived SCFA that is abundant in the healthy gut (Fig. 1). As a result, colonocytes consume high levels of oxygen (O_2_), which in turn drives the colonic surface to be hypoxic (Albenberg et al., 2014) and limits the diffusion of oxygen into the gut lumen. This mechanism maintains an anaerobic environment in the healthy colon, ensuring that beneficial obligate anaerobic bacteria dominate the intestinal microbial community.

**Figure:**
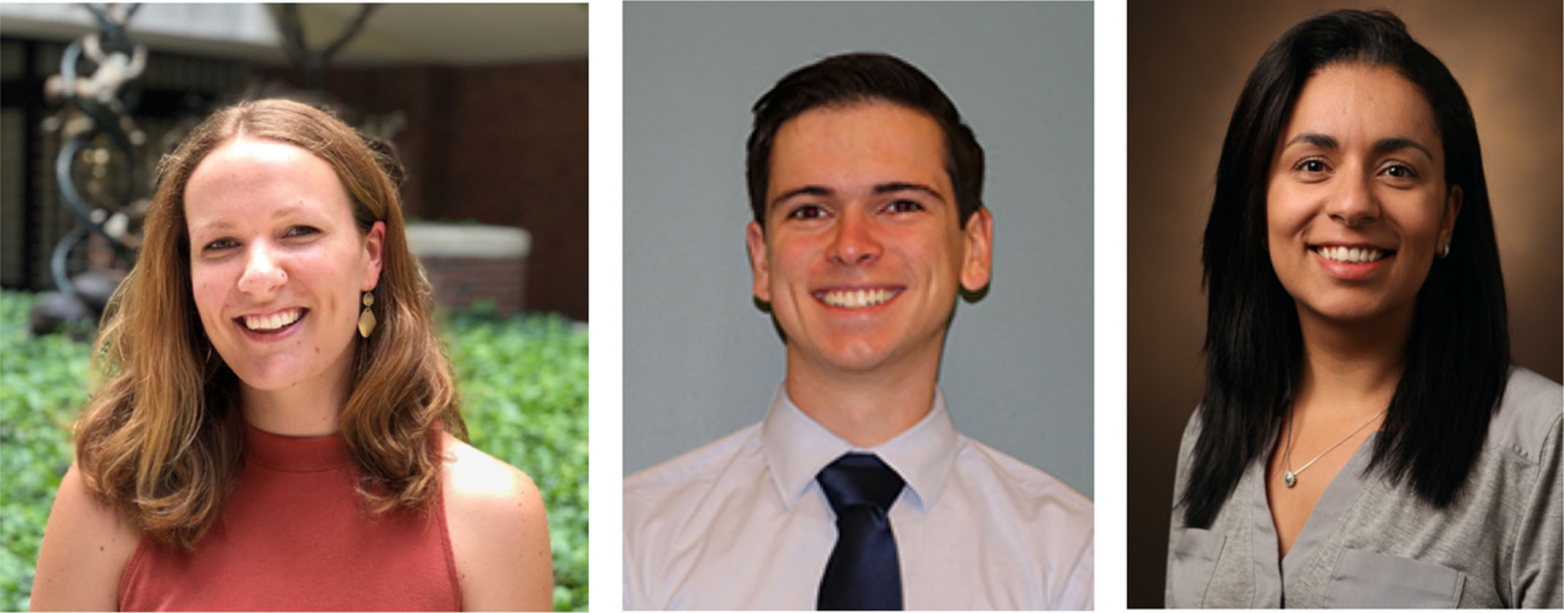
**Nora Foegeding, Zachary Jones and Mariana Byndloss** (from left to right)

Emerging research is providing early mechanistic insight into how environmental factors associated with a Westernized lifestyle disrupt the homeostatic mechanisms of the colonic epithelium, inducing the dysbiotic expansion of facultative anaerobes (Byndloss et al., 2017; Lee et al., 2020). For example, exposure to antibiotics has been shown to cause a reduction in the abundance of butyrate-producing microbes, leading to a decline in PPARG signaling in the colonic epithelium (Byndloss et al., 2017). The resulting decrease in mitochondrial respiration in colonocytes causes a corresponding increase in oxygen reaching the colonic lumen (Byndloss et al., 2017). As a result, and because of their ability – as facultative anaerobes – to utilize oxygen for aerobic respiration, pathogenic *E. coli* and *Salmonella* bloom in the gut following antibiotic treatment (Byndloss et al., 2017; Rivera-Chávez et al., 2016). Similarly, an obesogenic high-fat diet has recently been shown to impair mitochondrial function in the intestinal epithelium, causing disruption of the anaerobic environment of the healthy colonic lumen and driving the expansion of *E. coli* (Lee et al., 2020) (Fig. 1)*.* In addition to oxygen, environmental factors also influence the bioavailability of host-derived nitrate (NO_3_^−^) that is subsequently utilized by Enterobacteriaceae as a terminal electron acceptor to fuel anaerobic respiration (Byndloss et al., 2017; Spees et al., 2013; Winter et al., 2013). Indeed, Enterobacteriaceae and other anaerobic bacteria have the capacity to use an array of molecules aside from oxygen as terminal electron acceptors for respiration (Khademian and Imlay, 2017; Miller et al., 2020; Nguyen et al., 2020; Winter et al., 2010; Zhu et al., 2019). This suggests a common mechanism by which environmental factors spur dysbiosis in the colon is through a change in the abundance of alternative electron acceptors. These findings are particularly relevant in the context of CRC, as the use of antibiotics (Zhang et al., 2019), a high-fat diet (Willett et al., 1990) and accompanying diet-induced obesity have each been linked to an increased risk of CRC (Liu et al., 2019). Collectively, this new mechanistic insight leads to the idea that environmental factors associated with a Western lifestyle create a favorable environment for the growth of *pks^+^ E. coli,* a member of the microbiota that plays a key role in colorectal carcinogenesis (Fig. 1).

In the future, it will be important to determine whether alterations in colonocyte metabolism driven by environmental factors underlie the bloom of *pks*^+^ *E. coli* and other tumorigenic members of the gut microbiota. In addition, given the significant role of the intestinal epithelium in policing gut microbiota composition, it will be crucial to detail the mechanisms by which antibiotics, a high-fat diet and other environmental factors affect overall colonocyte metabolism. Last, while the focus of this Perspective is on the contribution of bacteria to CRC, environmental factors also influence the colonization of other microorganisms in the gastrointestinal tract, including fungi (Heisel et al., 2017) and viruses (Schulfer et al., 2020). Future research investigating the influence of these groups on CRC will undoubtedly yield insightful discoveries.

[…] environmental factors trigger dysbiosis by affecting intestinal epithelial cell metabolism, thus highlighting interactions between the colonic epithelium and the gut microbiota as new targets for CRC therapeutics

## The therapeutic potential of modulating gut microbiota

Modulating the gut microbiota is an emerging strategy for the prevention and treatment of CRC (Zhu et al., 2019; O'Keefe et al., 2015). Collectively, the examples discussed above convey the new idea that environmental factors trigger dysbiosis by affecting intestinal epithelial cell metabolism, thus highlighting interactions between the colonic epithelium and the gut microbiota as new targets for CRC therapeutics (Cevallos et al., 2021).

To better inform on therapeutic interventions targeting host-microbe interactions in the gut, a main aim of future research should be to further detail the mechanisms by which environmental factors and gut microbiota – both individually and in combination – influence gastrointestinal health. Which of the many members of the gut microbiota have key roles in regulating local conditions and affecting cellular processes in ways that have a significant influence on colorectal carcinogenesis? How does the adoption of a Westernized diet, a trend linked to the increasing incidence of early-onset CRC, affect the long-term health of the intestinal epithelium? How does the abundance of harmful and helpful microbial products change in response to dietary factors and antibiotic exposure? What are the consequences of a concurrent increase in inflammatory tone and harmful microbial products, and is it sufficient to spur carcinogenesis? These are just a few of the many unanswered questions that should be addressed as we endeavor to manage gastrointestinal health by modulating the gut microbiome.
